# The complete chloroplast genome of an Antarctic moss, *Ptychostomum pseudotriquetrum* (Hedw.) J.R.Spence & H.P.Ramsay (Bryaceae), and phylogenetic analysis

**DOI:** 10.1080/23802359.2024.2384580

**Published:** 2024-09-01

**Authors:** Sang Ho Lee

**Affiliations:** Department of Biological Sciences, Mokwon University, Daejeon, Republic of Korea

**Keywords:** Antarctic, Bryaceae, chloroplast genome, *Ptychostomum*

## Abstract

*Ptychostomum pseudotriquetrum* (Hedw.) J.R.Spence & H.P.Ramsay (Bryaceae) is a bipolar and one of the most widespread species within Antarctica, exhibiting a ubiquitous presence along the Antarctic Peninsula. This study analyzed its chloroplast genome, which is 123,172 bp in length, and consists of 82 protein-coding genes, four ribosomal RNA genes, and 31 transfer RNA genes. A phylogenetic tree, constructed using 58 conserved orthologous protein-coding genes from 19 complete chloroplast genomes of the class Bryopsida, confirmed that *P. pseudotriquetrum* belongs to clade Bryaceae. Within this clade, *P. pseudotriquetrum* diverged from the clade containing *Anomobryum gemmigerum* and *Bryum argenteum*. This study contributes to enriching chloroplast genome resources for the family Bryaceae and the genus *Ptychostomum*. Such advancement could facilitate future genetic investigations aimed at conserving and exploiting Antarctic bryophytes.

## Introduction

*Ptychostomum pseudotriquetrum* (Hedw.) J.R.Spence & H.P.Ramsay, known by its homotypic synonyms *Bryum pseudotriquetrum* (Hedw.) P.Gaertn., B.Mey. & Scherb. and *Plagiobryum pseudotriquetrum* (Hedw.) N.Pedersen, belongs to the family Bryaceae (Holyoak and Pedersen 2007). While *P. pseudotriquetrum* is often regarded as a cosmopolitan species, it is more precisely described as a bipolar moss with intermediate stations found at high elevations in tropical Africa. Within Antarctica, it ranks among the most widespread moss species, exhibiting a ubiquitous presence throughout the South Orkney Islands and South Shetland Islands archipelagos, with varied distribution along the Antarctic Peninsula. Furthermore, this species is a common component of the Antarctic herb tundra formation, often coexisting with the only two species of vascular plants native to this biome, *Deschampsia antarctica* and *Colobanthus quitensis* (Ochyra et al. 2008).

It typically thrives in well-drained, sheltered habitats, predominantly on sloping ground at low elevations in coastal areas. In Antarctica, certain populations of *P. pseudotriquetrum* are submerged in lakes, occasionally reaching considerable depths. Records of *P. pseudotriquetrum* growth have been documented in freshwater lakes, such as Radok Lake in continental Antarctica, where it has been observed at depths of up to 81 m (Wagner and Seppelt 2006).

Given the morphological variations observed as inconsistent phenotypes of *P. pseudotriquetrum*, taxonomic inference is derived not only from the assessment of morphological traits but also from phylogenetic analyses of DNA sequences across various phenotypes of this species (Holyoak and Hedenäs 2006). Furthermore, investigations into the flavonoid composition of *P. pseudotriquetrum* (referred to as *Bryum algens*) by Webby et al. (1996) have uncovered multiple chemotypes within this species. The current study aims to enhance the understanding of the taxonomic position of *P. pseudotriquetrum* and its evolution within clade Bryaceae by analyzing its chloroplast genome, a subject not previously explored.

## Materials and methods

**Figure 1.**
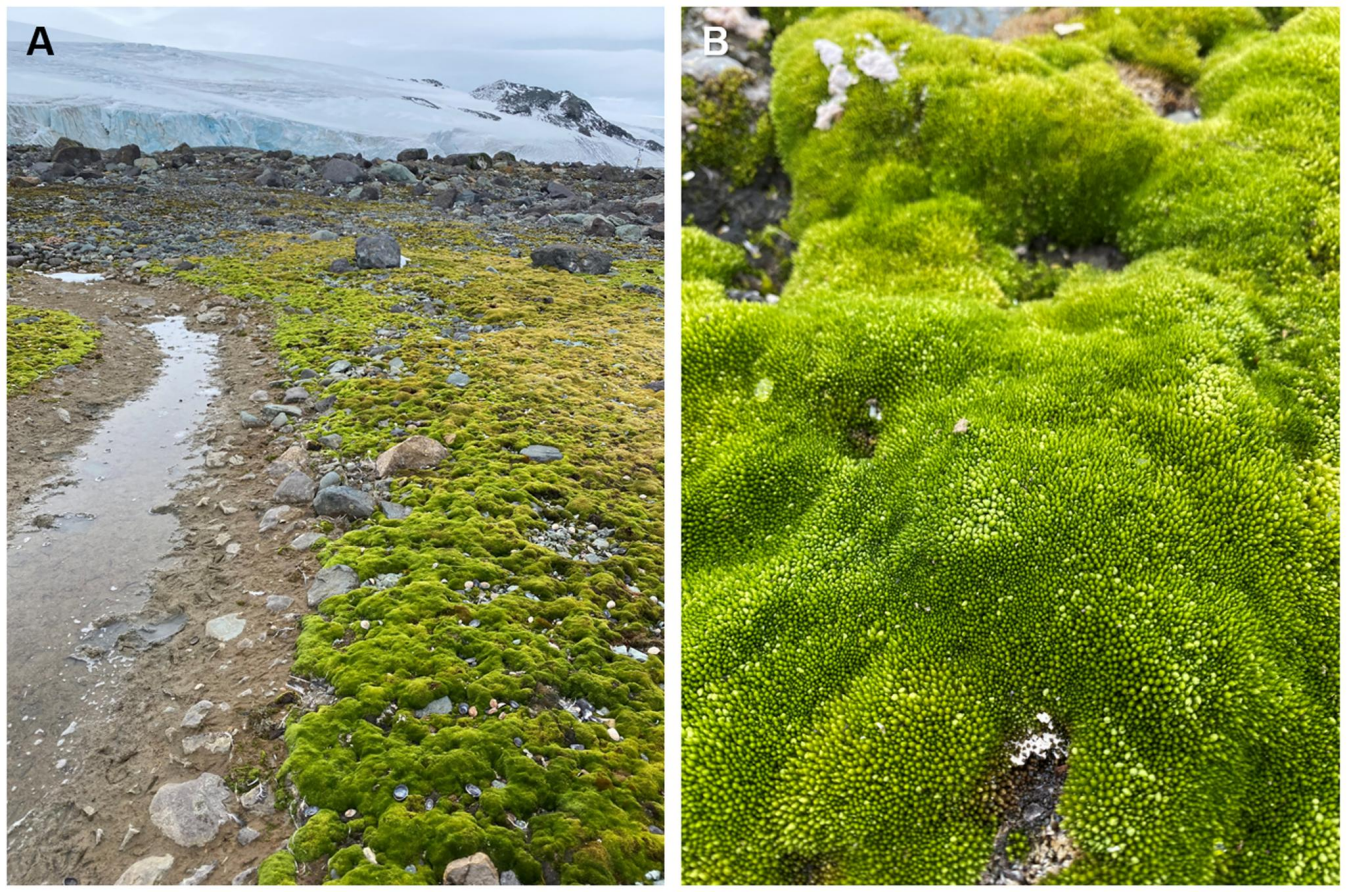
Reference images of *Ptychostomum pseudotriquetrum*. It is one of the most widespread moss species found in Antarctica, typically growing in moist and relatively sheltered locations such as the edges of meltwater streams (A). The characteristic morphological feature is the dense extensive carpets of small-sized plants with whitish uppermost leaves (B). Photographs were taken by Hyoungseok Lee in Barton Peninsula, King George Island (62°13′49.9″ S; 58°42′40.3″ W) on 18 February 2022.

The *Ptychostomum pseudotriquetrum* sample was collected in February 2022 by Hyoungseok Lee from a population growing under natural conditions near the King Sejong Antarctic Station (62°13′49.9″ S; 58°42′40.6″ W) on Barton Peninsula of King George Island (Figure 1). A specimen was deposited at the Korea Polar Research Institute (KOPRI) Herbarium (https://kvh.kopri.re.kr, Han-Gu Choi, hchoi82@kopri.re.kr) under voucher number KOPRI-MO00904 and identified as *P. pseudotriquetrum* by Hyoungseok Lee.

Total genomic DNA was purified using a BiomedicVR Plant gDNA Extraction Kit (Biomedic Co., Ltd., Bucheon, South Korea) according to the manufacturer’s protocol. Genomic library construction was performed using a TruSeq PCR Free DNA Sample Prep Kit (Illumina, San Diego, CA), and paired-end whole-genome sequences were produced using the Illumina NovaSeq platform (San Diego, CA). A total of 14,813,700 filtered reads with a mean length of 145.0 bp were obtained, and the total read length of raw data was 2.4 Gb. The raw sequencing data underwent trimming followed by *de novo* assembly with CLC Assembly Cell v4.2.1 (CLC bio, Aarhus, Denmark). Among the assembled contigs, chloroplast genome sequences were retrieved, aligned, and merged into a single sequence using the *Sanionia uncinata* chloroplast genome sequence as a reference (Park et al. 2018). The assembly underwent manual verification to finalize it, and any sequence errors were corrected through read mapping against the assembled contig. To verify the accuracy of the assembly, trimmed raw sequence data were mapped to the assembled chloroplast genome and the average coverage depth was ×720.6 (Supplementary Figure 1).

Genes were annotated using the GeSeq program (Tillich et al. 2017) with reported Bryaceae chloroplast genomes (ON310499, MW602653, and MW147233) as references, and manually curated using the Artemis program (Carver et al. 2012). The complete chloroplast genome of *P. pseudotriquetrum* has been deposited in GenBank under accession number OR911497. A circular genome map was generated using CPGView (Liu et al. 2023). From a total of 19 chloroplast genome sequences, including *P. pseudotriquetrum* nucleotide sequences of 58 conserved orthologous protein-coding genes (*acc*D, *atp*A, *atp*B, *atp*E, *atp*F, *atp*H, *chl*B, *chl*N, *clp*P, *inf*A, *ndh*A, *ndh*B, *ndh*C, *ndh*D, *ndh*E, *ndh*F, *ndh*J, *pet*B, *pet*D, *pet*G, *pet*L, *psa*A, *psa*B, *psa*C, *psa*J, *psb*A, *psb*B, *psb*C, *psb*D, *psb*E, *psb*F, *psb*H, *psb*J, *psb*L, *psb*M, *psb*T, *psb*Z, *rbc*L, *rpl*14, *rpl*16, *rpl*2, *rpl*20, *rpl*22, *rpl*23, *rpl*32, *rpl*33, *rpl*36, *rpo*C1, *rpo*C2, *rps*11, *rps*15, *rps*18, *rps*19, *rps*2, *rps*3, *rps*4, *rps*7, and *rps*8) were aligned using MAFFT version 7 (https://mafft.cbrc.jp/alignment/server/) (Katoh and Standley 2013), followed by the construction of a phylogenetic tree based on the maximum-likelihood method (ML; bootstrap repeat is 1000) based on the GTR + G + I model using MEGA11 (Tamura et al. 2021).

## Results

**Figure 2.**
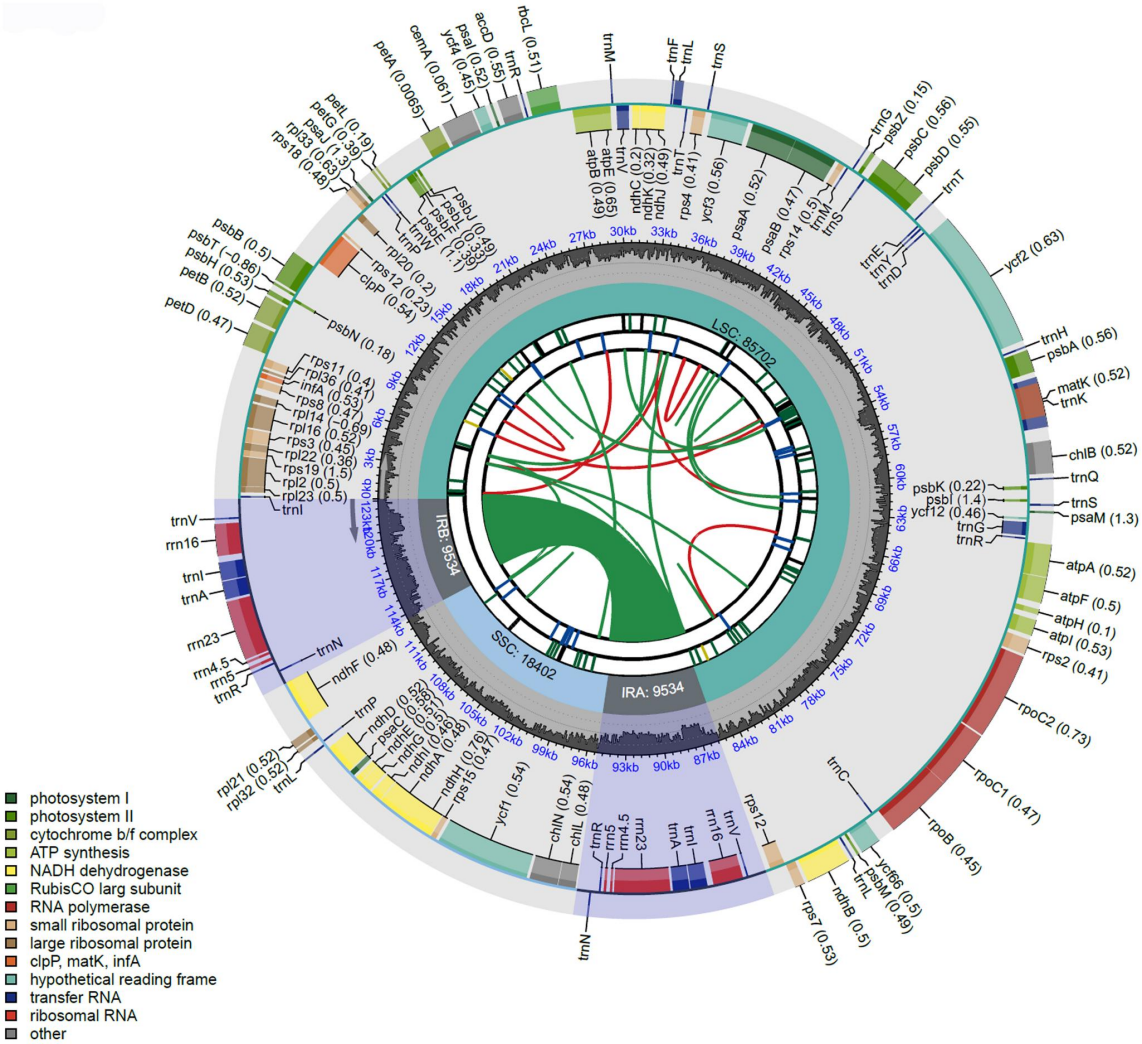
Map of the chloroplast genome of *Ptychostomum pseudotriquetrum*. Genes lying outside the outer circle are transcribed clockwise, while those inside the circle are transcribed counterclockwise. Genes belonging to different functional groups are color-coded. The innermost darker grey corresponds to GC content, while the lighter grey corresponds to at content. IR: inverted repeat; LSC: large single-copy region; SSC: small single-copy region.

**Figure 3.**
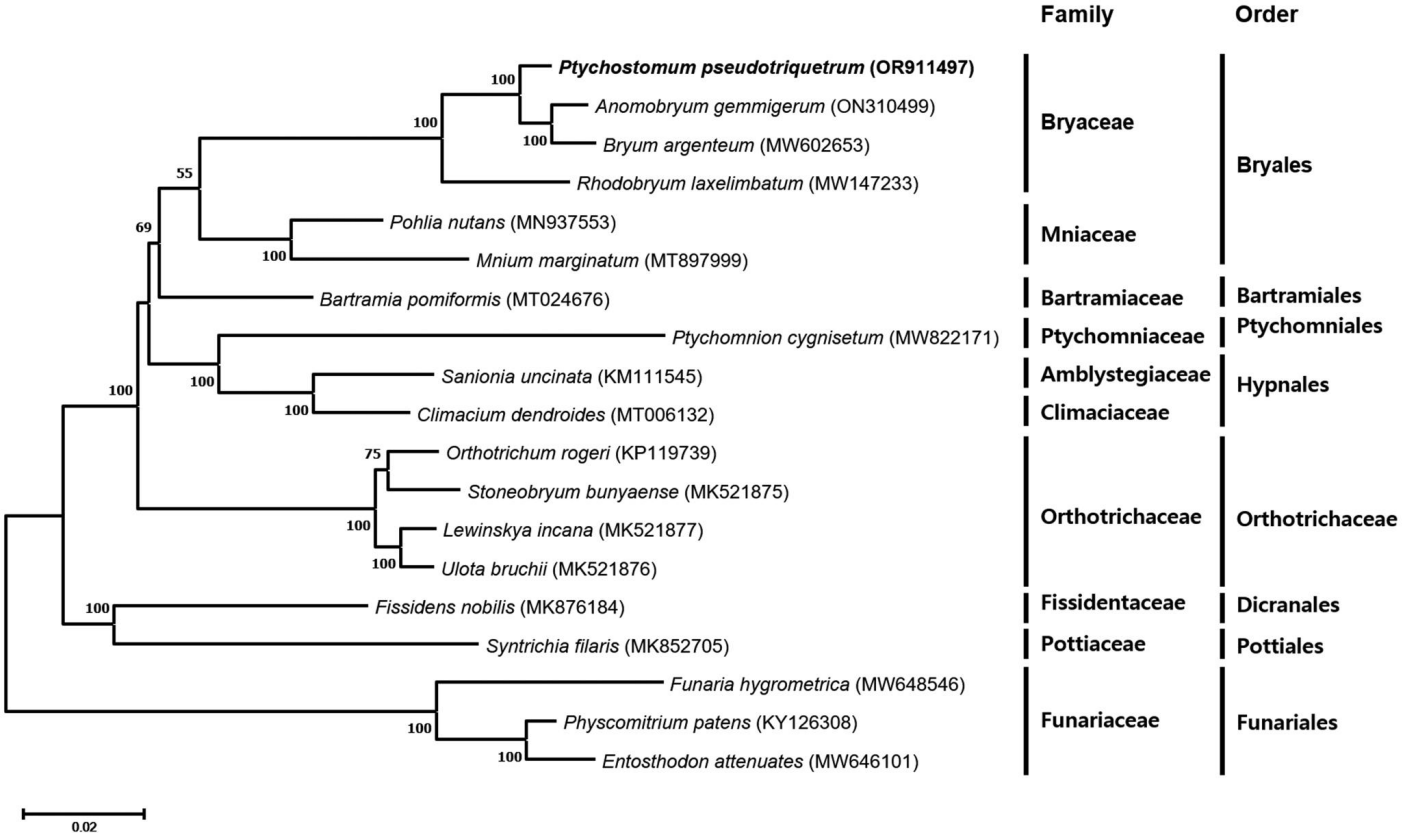
Maximum-likelihood phylogenetic tree of *Ptychostomum pseudotriquetrum* and its related species. Fifty-eight protein-coding sequences conserved in the chloroplast genomes of 19 species were multiple-aligned and used to generate a phylogenetic tree. The bootstrap support values (>50%) from 1000 replicates are indicated on the nodes. GenBank accession numbers of chloroplast genome sequences used for this tree are indicated within parentheses, and *P. pseudotriquetrum* analyzed in this study is represented by bold letters. The following sequences were used: *Ptychostomum pseudotriquetrum* (OR911497; this study), *Anomobryum gemmigerum* (ON310499), *Bryum argenteum* (MW602653) (Frangedakis et al. 2021), *Rhodobryum laxelimbatum* (MW147233) (Lubna et al. 2024), *Pohlia nutans* (MN937553) (Jin et al. 2020), *Mnium marginatum* (MT897999) (Shi et al. 2021), *Bartramia pomiformis* (MT024676) (Han, Park, et al. 2020), *Ptychomnion cygnisetum* (MW822171) (Frangedakis et al. 2021), *Sanionia uncinata* (KM111545) (Park et al. 2018), *Climacium dendroides* (MT006132) (Han, Choi, et al. 2020), *Orthotrichum rogeri* (KP119739) (Mizia et al. 2019), *Stoneobryum bunyaense* (MK521875) (Mizia et al. 2019), *Lewinskya incana* (MK521877) (Mizia et al. 2019), *Ulota bruchii* (MK521876) (Mizia et al. 2019), *Fissidens nobilis* (MK876184) (Kwon et al. 2019), *Syntrichia filaris* (MK852705) (Kim et al. 2019), *Funaria hygrometrica* (MW648546) (Frangedakis et al. 2021), *Physcomitrium patens* (KY126308) (Sugiura et al. 2003), *Entosthodon attenuatus* (MW646101) (Frangedakis et al. 2021). *Funaria hygrometrica*, *Physcomitrium patens*, and *Entosthodon attenuatus* were used as outgroup taxa. Scale bar refers to a phylogenetic distance of 0.05 nucleotide substitutions per site.

The complete chloroplast genome sequence of *P. pseudotriquetrum* is 123,172 bp in length with GC content of 28.08%, composed of a large single-copy (LSC) region of 85,702 bp, a small single-copy (SSC) region of 18,402 bp, and 9534 bp of paired inverted repeat (IR) regions. In total, 117 genes were annotated, comprising 82 protein-coding genes, 31 tRNA genes, and four rRNA genes (Figure 2). There were 15 genes (*atp*F, *ndh*A, *ndh*B, *pet*B, *pet*D, *rpl*2, *rpl*16, *rpo*C1, *ycf*66, *trn*A-UGC, *trn*G-UCC, *trn*I-GAU, *trn*K-UUU, *trn*L-UAA, and *trn*V-UAC) containing one intron and three genes (*clp*P, *rps*12, and *ycf*3) having two introns. Ten cis-spliced genes (*atp*F, *clp*P, *ndh*A, *ndh*B, *pet*B, *pet*D, *rpl*2, *rpl*16, *rpo*C1, and *ycf*3) and one trans-spliced gene (*rps*12) were verified to be corrected and annotated with multiple sequence alignment (Supplementary Figure 2). To evaluate the evolutionary relationships, 19 chloroplast genome sequences including four complete chloroplast genomes of Bryaceae were used. As shown in Figure 3, Bryaceae and Mniaceae are clustered together to form the order Bryales, which is consistent with a previous study on the phylogenetics of the Bryophyte (Cole et al. 2019).

## Discussion and conclusions

In this study, the first chloroplast genome of *P. pseudotriquetrum* was assembled and annotated. By examining the genetic variation of the chloroplast genomes, taxonomic ambiguities can be resolved and evolutionary relationships within the Bryaceae can be understood. In the ML phylogenetic tree of *P. pseudotriquetrum* and its related species, the poorly supported node of the order Bryales (bootstrap value = 55) was divided into two well-supported clades: Bryaceae and Mniaceae. *Rhodobryum laxelimbatum* branched from the remaining taxa at the first node of Bryaceae. At the subsequent node, *P. pseudotriquetrum* diverged from the clade containing *Anomobryum gemmigerum* and *Bryum argenteum*. This taxonomic placement of *P. pseudotriquetrum* is consistent with previous studies on Bryaceae phylogeny, including *rps4* molecular phylogeny (Cannone et al. 2024), consensus analysis of molecular (four different chloroplast sequences), morphological, and indel data (Pedersen et al. 2003), and combined molecular phylogeny using nrDNA (ITS) and cpDNA (*rps4*) (Wang and Zhao 2009).

In conclusion, this study has contributed to chloroplast genome resources for the family Bryaceae and the genus *Ptychostomum*. These advancements are expected to facilitate future genetic investigations aimed at the conservation and exploitation of Antarctic bryophytes. As sequencing technologies continue to advance rapidly, the availability of additional genomic resources is anticipated to unveil more detailed phylogenetic relationships within Bryaceae.

## Supplementary Material

Supple Figures v1.pdf

## Data Availability

The genome sequence data that support the findings of this study are openly available in GenBank of NCBI at https://www.ncbi.nlm.nih.gov under the accession no. OR911497. The associated BioProject, SRA, and Bio-Sample numbers are PRJNA1049510, SRR27118464, and SAMN38700328, respectively.

## References

[CIT0001] Cannone N, Vanetti I, Convey P, Sancho LG, Zaccara S. 2024. Molecular analyses support revision of species diversity of the moss genus *Bryum* in Antarctica. Bot J Linn Soc. 205(1):15–25. doi:10.1093/botlinnean/boad070.

[CIT0002] Carver T, Harris SR, Berriman M, Parkhill J, McQuillan JA. 2012. Artemis: an integrated platform for visualization and analysis of high-throughput sequence-based experimental data. Bioinformatics. 28(4):464–469. doi:10.1093/bioinformatics/btr703. 22199388 PMC3278759

[CIT0003] Cole TC, Hilger HH, Goffinet B. 2019. Bryophyte phylogeny poster (BPP). PeerJ Prepr. 7:e27571v3.

[CIT0004] Frangedakis E, Guzman-Chavez F, Rebmann M, Markel K, Yu Y, Perraki A, Tse SW, Liu Y, Rever J, Sauret-Gueto S, et al. 2021. Construction of DNA tools for hyperexpression in *Marchantia* chloroplasts. ACS Synth Biol. 10(7):1651–1666. doi:10.1021/acssynbio.0c00637. 34097383 PMC8296666

[CIT0005] Han YD, Choi Y, Park S, Park YS, Yoon YJ. 2020. The complete chloroplast genome of a moss Korea *Climacium dendroides* (Hedw.) F. Weber & D. Mohr. Mitochondrial DNA B Resour. 5(2):1200–1201. doi:10.1080/23802359.2020.1731362. 33366911 PMC7510824

[CIT0006] Han YD, Park S, Choi Y, Kim S, Yoon YJ. 2020. The complete chloroplast genome of a moss Korea *Bartramia pomiformis* Hedw. Mitochondrial DNA B Resour. 5(2):1687–1688. doi:10.1080/23802359.2020.1748536. PMC7510824 33366911

[CIT0007] Holyoak DT, Hedenäs L. 2006. Morphological, ecological and molecular studies of the intergrading taxa *Bryum neodamense* and *B. pseudotriquetrum* (Bryopsida: Bryaceae). J Bryol. 28(4):299–311. doi:10.1179/174328206X136304.

[CIT0008] Holyoak DT, Pedersen N. 2007. Conflicting molecular and morphological evidence of evolution within the Bryaceae (Bryopsida) and its implications for generic taxonomy. J Bryol. 29(2):111–124. doi:10.1179/174328207X189198.

[CIT0009] Jin Q, Zhang L, Li D, He Y, Qu C, Miao J. 2020. Characterization of the complete chloroplast genome of the *Pohlia nutans* M211 from Antarctica. Mitochondrial DNA B Resour. 5(1):1096–1097. doi:10.1080/23802359.2020.1721368. 33366890 PMC7748587

[CIT0010] Katoh K, Standley DM. 2013. MAFFT multiple sequence alignment software version 7: improvements in performance and usability. Mol Biol Evol. 30(4):772–780. doi:10.1093/molbev/mst010. 23329690 PMC3603318

[CIT0011] Kim SC, Byun MY, Kim JH, Lee H. 2019. The complete chloroplast genome of an Antarctic moss *Syntrichia filaris* (Müll. Hal.) R.H. Zander. Mitochondrial DNA B Resour. 4(2):2303–2304. 33365516 10.1080/23802359.2019.1627945 PMC7687419

[CIT0012] Kwon W, Min J, Xi H, Park J. 2019. The complete chloroplast genome of *Fissidens nobilis* Griff. (Fissidentaceae, Bryophyta). Mitochondrial DNA B Resour. 4(2):2225–2226. doi:10.1080/23802359.2019.1623120. 33365485 PMC7687450

[CIT0013] Liu S, Ni Y, Li J, Zhang X, Yang H, Chen H, Liu C. 2023. CPGView: a package for visualizing detailed chloroplast genome structures. Mol Ecol Resour. 23(3):694–704. doi:10.1111/1755-0998.13729. 36587992

[CIT0014] Lubna, Asaf S, Jan R, Asif S, Bilal S, Khan AL, Kim K-M, Lee I-J, Al-Harrasi A. 2024. Plastome diversity and evolution in mosses: insights from structural characterization, comparative genomics, and phylogenetic analysis. Int J Biol Macromol. 257(Pt 2):128608. doi:10.1016/j.ijbiomac.2023.128608. 38065441

[CIT0015] Mizia P, Myszczyński K, Ślipiko M, Krawczyk K, Plášek V, Szczecińska M, Sawicki J. 2019. Comparative plastomes analysis reveals the first infrageneric evolutionary hotspots of *Orthotrichum* s.l. (Orthotrichaceae, Bryophyta). Turk J Bot. 43(4):444–457. doi:10.3906/bot-1811-13.

[CIT0016] Ochyra R, Bednarek-Ochyra H, Smith RIL. 2008. Illustrated moss flora of Antarctica. New York: Cambridge University Press.

[CIT0017] Park M, Park H, Lee H, Lee BH, Lee J. 2018. The complete plastome sequence of an Antarctic bryophyte *Sanionia uncinata* (Hedw.) Loeske. Int J Mol Sci. 19(3):709. doi:10.3390/ijms19030709. 29494552 PMC5877570

[CIT0018] Pedersen N, Cox CJ, Hedenäs L. 2003. Phylogeny of the moss family Bryaceae inferred from chloroplast DNA sequences and morphology. Syst Bot. 28(3):471–482.

[CIT0020] Shi S, Li S, Zhang S, Shen F, Niu J, Li L, Zhao J. 2021. The complete chloroplast genome of *Mnium marginatum* (With.) P. Beauv. Mitochondrial DNA B Resour. 6(3):837–839. doi:10.1080/23802359.2021.1884025. 33763597 PMC7954419

[CIT0021] Sugiura C, Kobayashi Y, Aoki S, Sugita C, Sugita M. 2003. Complete chloroplast DNA sequence of the moss *Physcomitrella patens*: evidence for the loss and relocation of *rpoA* from the chloroplast to the nucleus. Nucleic Acids Res. 31(18):5324–5331. doi:10.1093/nar/gkg726. 12954768 PMC203311

[CIT0022] Tamura K, Stecher G, Kumar S. 2021. MEGA11: molecular evolutionary genetics analysis version 11. Mol Biol Evol. 38(7):3022–3027. doi:10.1093/molbev/msab120. 33892491 PMC8233496

[CIT0023] Tillich M, Lehwark P, Pellizzer T, Ulbricht-Jones ES, Fischer A, Bock R, Greiner S. 2017. GeSeq-Versatile and accurate annotation of organelle genomes. Nucleic Acids Res. 45(W1):W6–W11. doi:10.1093/nar/gkx391. 28486635 PMC5570176

[CIT0024] Wagner B, Seppelt RD. 2006. Deep water occurrence of the moss *Bryum pseudotriquetrum* in Radok Lake, Amery Oasis, East Antarctica. Polar Biol. 29(9):791–795. doi:10.1007/s00300-006-0116-7.

[CIT0025] Wang CY, Zhao JC. 2009. Phylogeny of *Ptychostomum* (Bryaceae, Musci) inferred from sequences of nuclear ribosomal DNA internal transcribed spacer (ITS) and chloroplast *rps4*. J Syst Evol. 47(4):311–320. doi:10.1111/j.1759-6831.2009.00033.x.

[CIT0026] Webby RF, Markham KR, Smith RI. 1996. Chemotypes of the Antarctic moss *Bryum algens* delineated by their flavonoid constituents. Biochem Syst Ecol. 24(5):469–475. doi:10.1016/0305-1978(96)88877-6.

