# Supplementary material for: The complete chloroplast genome of an Antarctic moss, Ptychostomum pseudotriquetrum (Hedw.) J.R.Spence & H.P.Ramsay (Bryaceae), and phylogenetic analysis
Source: Mitochondrial DNA B Resour. 2024 Sep 1;9(9):1147–51. doi: 10.1080/23802359.2024.2384580 (PMC11370667; doi:10.1080/23802359.2024.2384580)
Supplement: Supple Figures v1.pdf [file TMDN_A_2384580_SM6954.pdf]

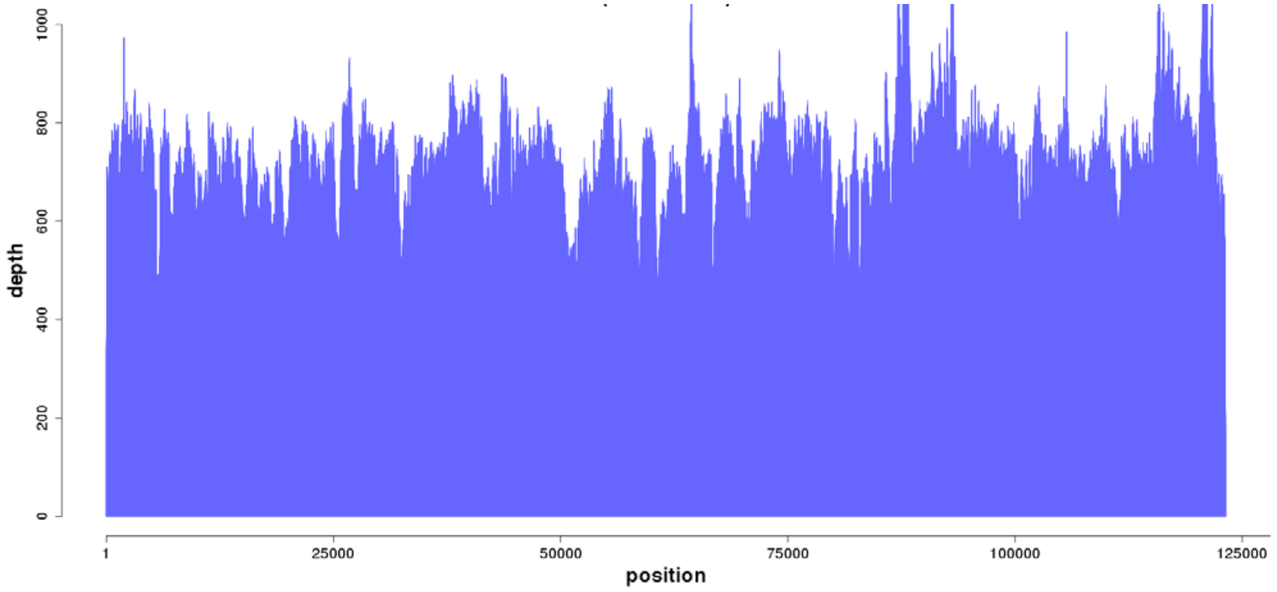

Supplementary Figure 1. Coverage depth figure of the *Ptychostomum pseudotriquetrum* plastome. The horizontal coordinate is the base of the plastome and the vertical coordinate is the depth of sequencing corresponding to that base.

A

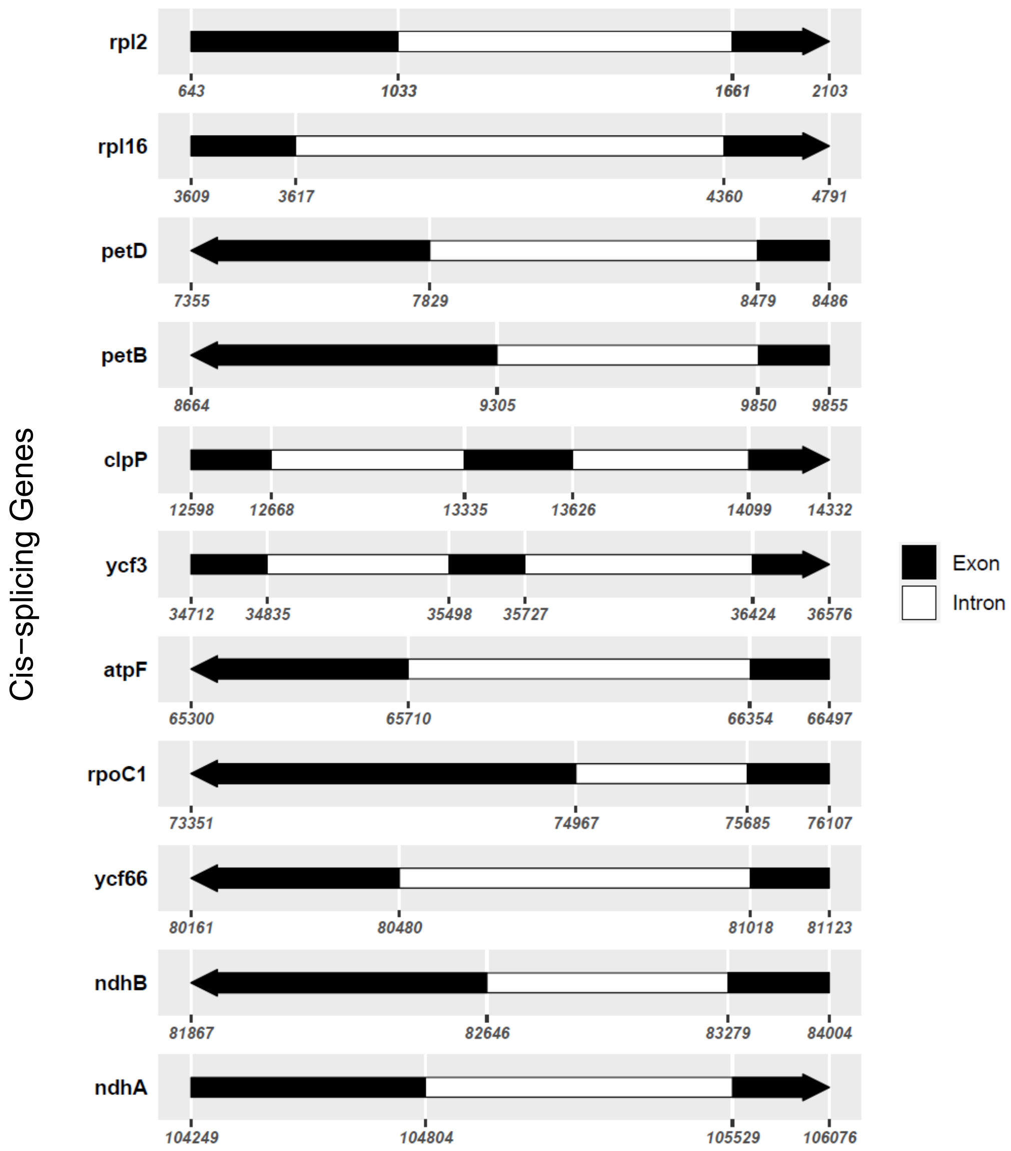

B

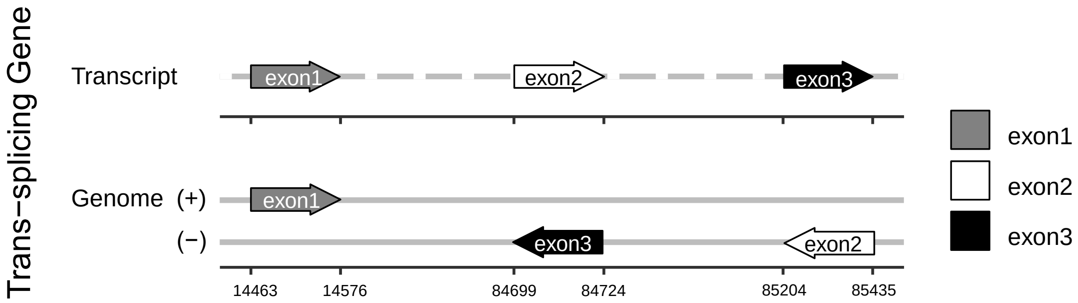

Supplementary Figure 2. Schematic map of the cis-splicing genes (A) and trans-splicing gene *rps12* (B) in the plastome of *Ptychostomum pseudotriquetrum*. The exons are shown in black; the introns are shown in white for (A). The arrow indicates the sense direction of the gene. The map was generated using CPGview
